# Evaluation of the prognostic value of CBXs in gastric cancer patients

**DOI:** 10.1038/s41598-021-91649-7

**Published:** 2021-06-11

**Authors:** Mengya He, Limin Yue, Haiyan Wang, Feiyan Yu, Mingyang Yu, Peng Ni, Ke Zhang, Shuaiyin Chen, Guangcai Duan, Rongguang Zhang

**Affiliations:** 1 Department of Epidemiology, College of Public Health, Zhengzhou University, No.100 Kexue Avenue, Zhengzhou, 450001 China; 2 Department of Epidemiology, College of Public Health, Hainan Medical University, Longhua District, No.3 Xueyuan Road, Haikou, 570216 China; 3 Department of Experimentation Center, College of Public Health, Zhengzhou University, No.100 Kexue Avenue, Zhengzhou, 450001 China

**Keywords:** Gastrointestinal cancer, Tumour biomarkers

## Abstract

Chromobox (CBX) proteins were suggested to exert epigenetic regulatory and transcriptionally repressing effects on target genes and might play key roles in the carcinogenesis of a variety of carcinomas. Nevertheless, the functions and prognostic significance of CBXs in gastric cancer (GC) remain unclear. The current study investigated the roles of CBXs in the prognosis of GC using the Oncomine, The Gene Expression Profiling Interactive Analysis (GEPIA), UALCAN, The Cancer Genome Atlas (TCGA), and cBioPortal databases. CBX1/2/3/4/5 were significantly upregulated in GC tissues compared with normal tissues, and CBX7 was downregulated. Multivariate analysis showed that high mRNA expression levels of CBX3/8 were independent prognostic factors for prolonged OS in GC patients. In addition, the genetic mutation rate of CBXs was 37% in GC patients, and genetic alterations in CBXs showed no association with OS or disease-free survival (DFS) in GC patients. These results indicated that CBX3/8 can be prognostic biomarkers for the survival of GC patients.

## Introduction

Gastric cancer (GC) is globally one of the three leading causes of cancer-associated mortality^1,2^. In 2015, GC was the third most frequently diagnosed carcinoma among Chinese males, while it was the fourth most frequently diagnosed carcinoma among Chinese females^3^. Although treatments such as surgery, radiotherapy, chemotherapy and others have been improved in recent years, the overall clinical outcomes of GC patients are still not ideal, and the 5-year survival rate is less than 30% because most patients are diagnosed with metastatic or unresectable GC^4–6^.

The polycomb group (PcG) protein family involves a group of epigenetic inhibitory proteins that are modified by chromatin histones^7,8^. The two characterized complexes of PcG proteins and the polycomb repressive complex 1/2 (PRC1/2) are essential for maintaining the stemness of embryonic and mature stem cells^9^. In PRC1, chromobox (CBX) family proteins are crucial components of the epigenetic regulation complexes that participate in the tumorigenesis and progression of many carcinomas^9–11^. To date, eight CBX family proteins have been identified in the human genomes^12^. According to the molecular structure of CBX family proteins, they can be divided into two groups: the heterochromatin protein 1 (HP1) group (including CBX1, CBX3, and CBX5) and the Pc group (including CBX2, CBX4, CBX6, CBX7, and CBX8)^13^. In mammals, the Pc group contains only a conserved N-terminal chromodomain that participate in the formation of PRC1 and stabilizes the binding of PRC1 to chromatin, while the HP1 group consists of an N-terminal domain and a C-terminal chromoshadow domain^13–16^.

CBX family proteins have pathogenic effects on a variety of carcinomas. Studies have shown that CBX1 is upregulated in hepatocellular cancer (HCC), breast cancer (BC) and prostate cancer (PCa)^17–19^. Overexpression of CBX1 is correlated with poor recurrence-free survival in patients with breast cancer (BC)^17^. CBX2 overexpression is significantly correlated with progression and metastasis in many cancer types, especially BC^20^. In addition, overexpression of CBX3 has been found in colorectal cancer (CRC), lung adenocarcinoma (LUAD) and tongue squamous cell carcinoma (TSCC)^21–24^. Reduced CBX7 expression was shown to correlate with a high tumor grade in thyroid, pancreatic, breast, colon, and lung carcinomas^25–27^. Previous studies reported that CBX8 expression was elevated in HCC, and associated with adverse outcomes^28,29^. However, the roles of CBX family members in the development and progression of GC remain unclear. In the present study, the expression and mutations of different CBX family members and their relations with clinical parameters in GC patients were investigated, and furthermore, the relationship between CBXs and the prognosis in GC patients was also analyzed.

## Materials and methods

### Oncomine

The Oncomine database (www.oncomine.org) is a microarray database of tumor genes that contains 715 independent datasets and 86,733 samples. In this study, the differences in mRNA levels of CBXs between cancer samples and normal samples in a multitude of cancer types were analyzed using the Oncomine database. Every gene in the CBX family was analyzed using the following parameters: *P* value: 0.01, fold change: 2, gene rank: 10%, and data type: mRNA.

### GEPIA

The Gene Expression Profiling Interactive Analysis (GEPIA) database (http://gepia.cancer-pku.cn/) was used to explore the prognostic and clinicopathological significance of CBXs in GC. This database consists of two transcriptome databases, namely, The Cancer Genome Atlas (TCGA) and Genotype-tissue Expression (GTEx), and contains RNA sequencing expression data from 9736 carcinoma samples from 33 malignancies and 8587 normal samples^30^. In this study, relevant data from the Oncomine database were validated using the GEPIA database. The cutoff *P* value and fold change were as follows:|log_2_ (fold change)|1 and *P* value 0.01.

### UALCAN

UALCAN (http://ualcan.path.uab.edu) is an interactive web resource based on level 3 RNA-seq and clinical data from 31 cancer types from the TCGA database. In this study, UALCAN was used to analyze the associations of the mRNA expression levels of 8 CBX family members with clinicopathologic parameters in primary GC tissues. Differences in transcriptional expression were compared by Student’s *t* test, and *P* ˂ 0.05 was considered statistically significant.

### TCGA

The TCGA is a comprehensive and coordinated project designed to improve diagnostic methods and treatment standards and ultimately to prevent cancer. The sequencing and pathological data from more than 30 kinds of human tumors can be obtained from the TCGA. In the present analysis, the clinicopathological parameters of 373 GC patients and data on the mRNA expression levels of CBXs in 343 GC patients were downloaded from the TCGA database (https://www.cancer.gov/about-nci/organization/ccg/research/structural-genomics/tcga). A total of 38 GC patients were excluded because survival data were lacking. Finally, 335 GC patients with data on the mRNA expression levels of CBXs were included in the analysis. Clinical information, including sex, age, race, topography, lymph node status, and metastasis, is shown in Supplementary Table 1. Then, GSE84437 from the GEO database (https://www.ncbi.nlm.nih.gov/geo/) was used to validate the CBXs mRNA expression levels of CBXs related to the prognosis of GC patients.

### cBioPortal

cBioPortal (www.cbioportal.org) is an online open-access website resource for exploring, visualizing and analyzing multidimensional cancer genomics data. In this study, the genomic profiles of 8 CBX family members containing mutations and putative copy number alterations were obtained from GISTIC and those with an mRNA expression z-score (RNASeq V2 RSEM) of the threshold were analyzed. Genetic mutations in CBXs and their association with the OS and disease-free survival (DFS) of GC patients are displayed as Kaplan–Meier plots. The log-rank test was performed to identify significant differences between the survival curves. A *P* value < 0.05 was considered statistically significant.

### Statistical analysis

The associations of CBXs with the survival of GC patients were further analyzed with multivariate Cox regression using SPSS software (version 26.0; SPSS Inc., Chicago, IL, USA). *P* < 0.05 was considered statistically significant.

### Ethics statement

The study was approved by the Ethics Review Committee of Zhengzhou University. All the data were retrieved from the online public databases.

## Results

### Overexpression of different CBXs in GC patients

The expression levels of CBXs in cancer tissues and normal tissues were compared by using Oncomine. Significantly higher mRNA expression levels of CBX1/2/3/4 were found in GC tissues in multiple datasets. In the Cho dataset, CBX1 was overexpressed in GC tissues compared with normal tissues, with a fold change of 2.415 (*P* value = 4.52e−06)^31^. The DErrico dataset showed an increase in GC tissues, with a fold change of 2.116 (*P* value = 2.21e−13)^32^. There were increased mRNA expression levels of CBX2 in GC patients in the four datasets. In the Cho dataset, CBX2 was increased in diffuse gastric adenocarcinoma, with a fold change of 2.290 (*P* value = 6.01e−09)^31^, and in gastric mixed adenocarcinoma, with a fold change of 2.077 (*P* value = 3.75e−04)^31^. The DErrico dataset showed an increase in CBX2 mRNA in gastric intestinal-type adenocarcinoma tissues, with a fold change of 4.485 (*P* value = 1.79e−09)^32^. The Wang dataset showed that CBX2 mRNA was increased in GC tissues, with a fold change of 2.501 (*P* value = 0.002)^33^. In the DErrico dataset, CBX3 mRNA expression was upregulated in gastric intestinal-type adenocarcinoma tissues compared with normal tissues, with a fold change of 3.014 (*P* value = 6.64e−14)^32^. In addition, in the DErrico dataset, increased mRNA levels of CBX4 in diffuse gastric adenocarcinoma tissues (*P* value = 2.45e−05, fold change = 2.466)^32^ and gastric mixed adenocarcinoma tissues (*P* value = 2.29e−06, fold change = 3.314)^32^ were observed (Table 1).

**Table 1:** Significant changes of CBXs mRNA expression levels between GC tissues and normal gastric tissues (ONCOMINE).

CBXs	Types of GC vs. normal	Fold change	*t* test	*P* value	References
CBX1	Gastric adenocarcinoma vs. normal	2.415	6.913	4.52E−06	Cho Gastric^31^
	Gastric intestinal type adenocarcinoma vs. normal	2.116	10.157	2.21E−13	DErrico Gastric^32^
CBX2	Diffuse gastric adenocarcinoma vs. normal	2.290	6.862	6.01E−09	Cho Gastric^31^
	Gastric mixed adenocarcinoma vs. normal	2.077	4.349	3.75E−04	Cho Gastric^31^
	Gastric intestinal type adenocarcinoma vs. normal	4.485	7.310	1.70E−09	DErrico Gastric^32^
	Gastric cancer vs. normal	2.501	3.330	0.002	Wang Gastric^33^
CBX3	Gastric intestinal type adenocarcinoma vs. normal	3.014	9.795	6.64E−14	DErrico Gastric^32^
CBX4	Diffuse gastric adenocarcinoma vs. normal	2.466	4.862	2.45E−05	DErrico Gastric^32^
	Gastric mixed adenocarcinoma vs. normal	3.314	6.444	2.29E−06	DErrico Gastric^32^

*GC* gastric cancer, *CBX* chromobox.

**Figure 1:**
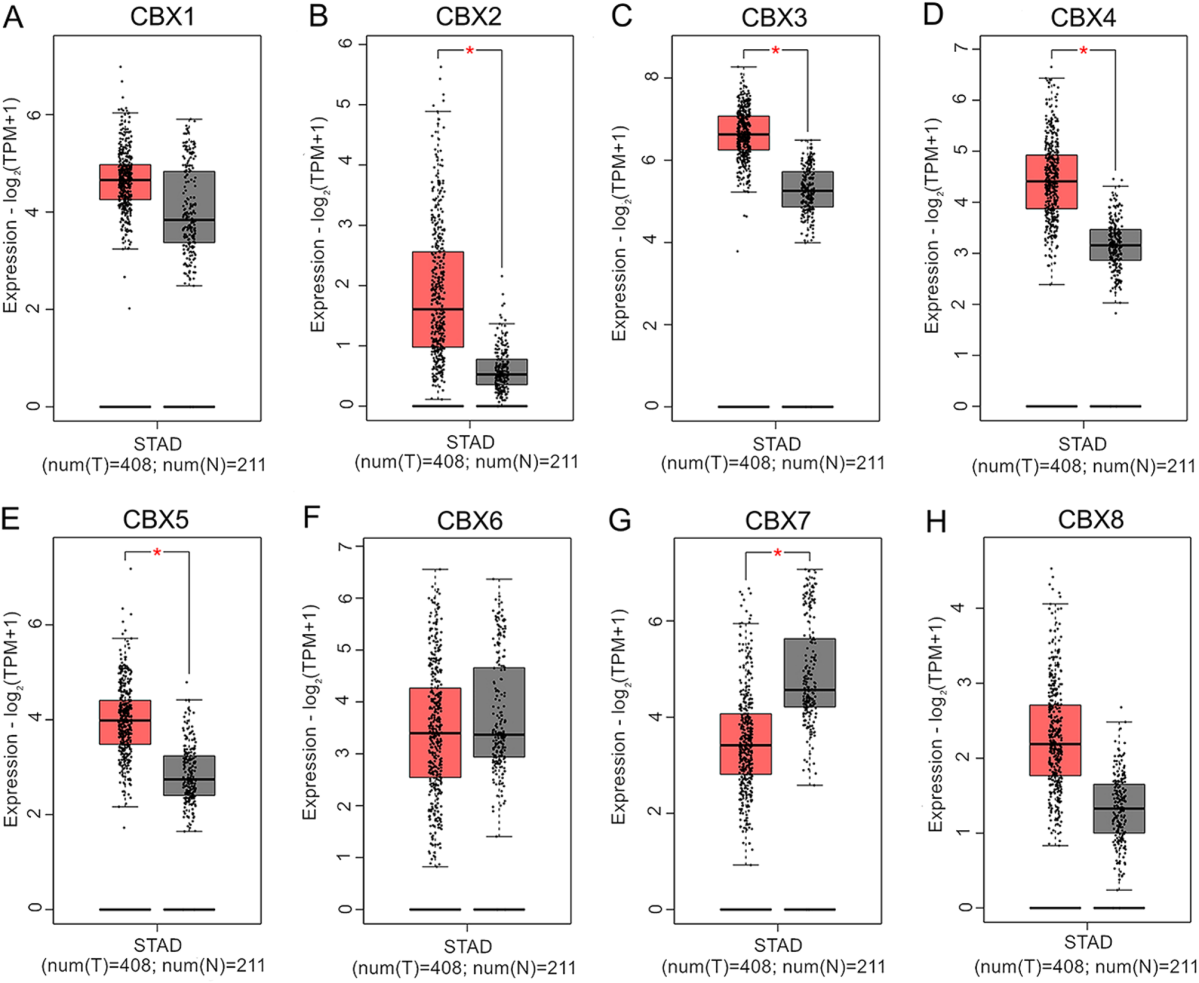
mRNA expression of CBXs in GC tissues and adjacent normal tissues (GEPIA). CBX2/3/4/5 mRNA expression was higher in primary GC tissues than in normal tissues (**B**–**E**). CBX7 mRNA expression was lower in primary GC tissues than in normal tissues (**G**). CBX1/6/8 mRNA expression was not significantly different between primary GC tissues and normal tissues (**A**,**F**,**H**). *P ˂ 0.01.

Moreover, the difference in the transcriptional expression of CBXs between GC tissues and normal tissues was further detected using the GEPIA dataset. The transcription levels of CBX2, CBX3, CBX4 and CBX5 were significantly enhanced (Fig. 1B–E), whereas the mRNA level of CBX7 was decreased in gastric cancer tissues compared with normal tissues (Fig. 1G). Additionally, no significant difference was detected in the mRNA levels of CBX1, CBX6 or CBX8 between GC tissues and normal tissues (Fig. 1A,F,H).

### Associations of the mRNA expression levels of CBXs with the clinicopathologic parameters of GC patients

**Figure 2:**
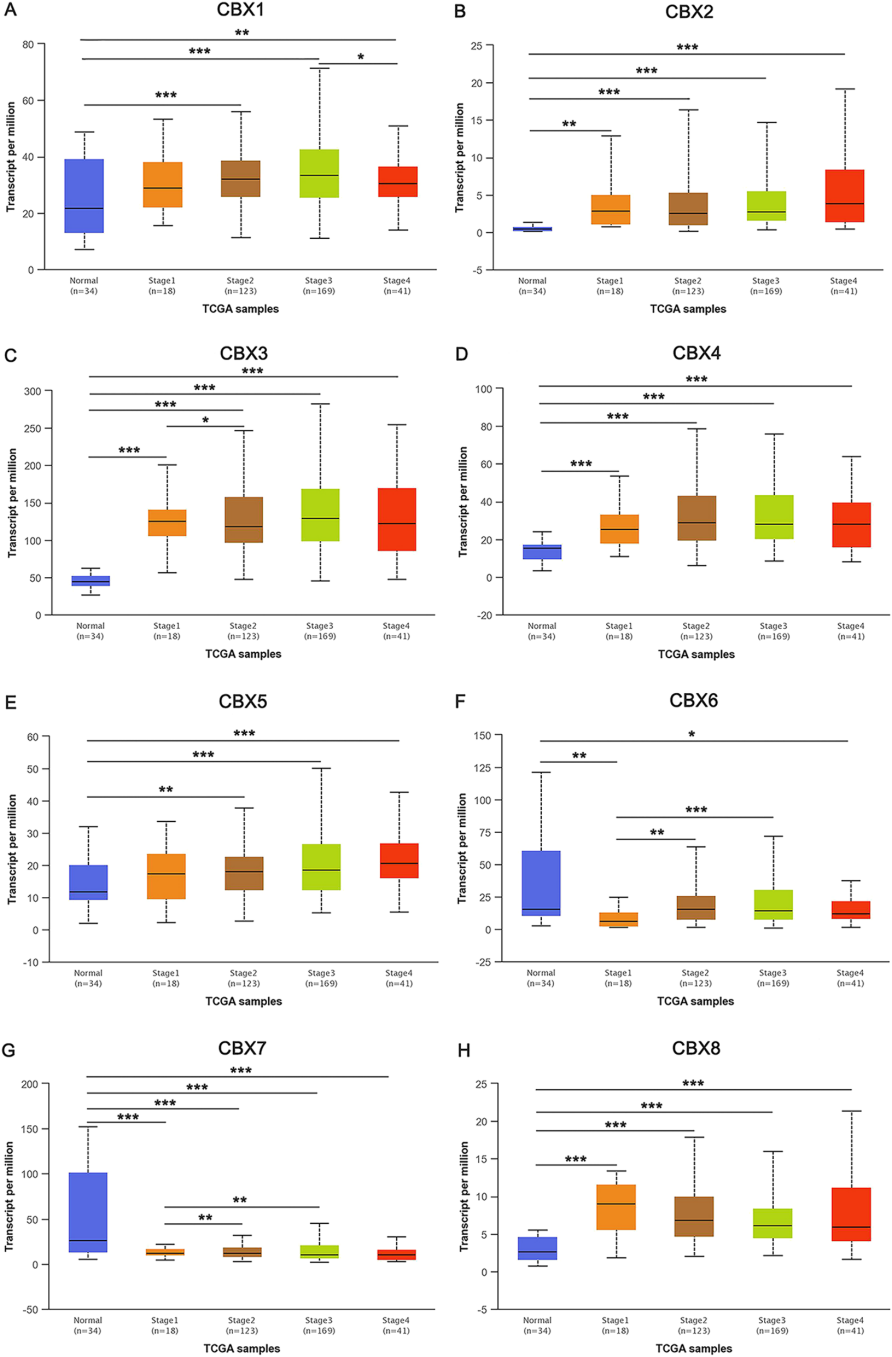
Relationship between the mRNA expression of CBXs and individual cancer stages of GC patients. The mRNA expression levels of 8 CBXs were remarkably related to individual cancer stages, and patients who were in more advanced stages tended to exhibit higher mRNA expression levels of CBXs. The mRNA expression levels of CBX1/3, CBX2/5, and CBX4/6 were the highest in stage 3 (**A**,**C**), stage 4 (**B**,**E**), and stage 2 (**D**,**F**), respectively. However, the mRNA expression levels of CBX7/8 were the highest in stage 1 (**G**,**H**). *P ˂ 0.05, **P ˂ 0.01, ***P ˂ 0.001.

**Figure 3:**
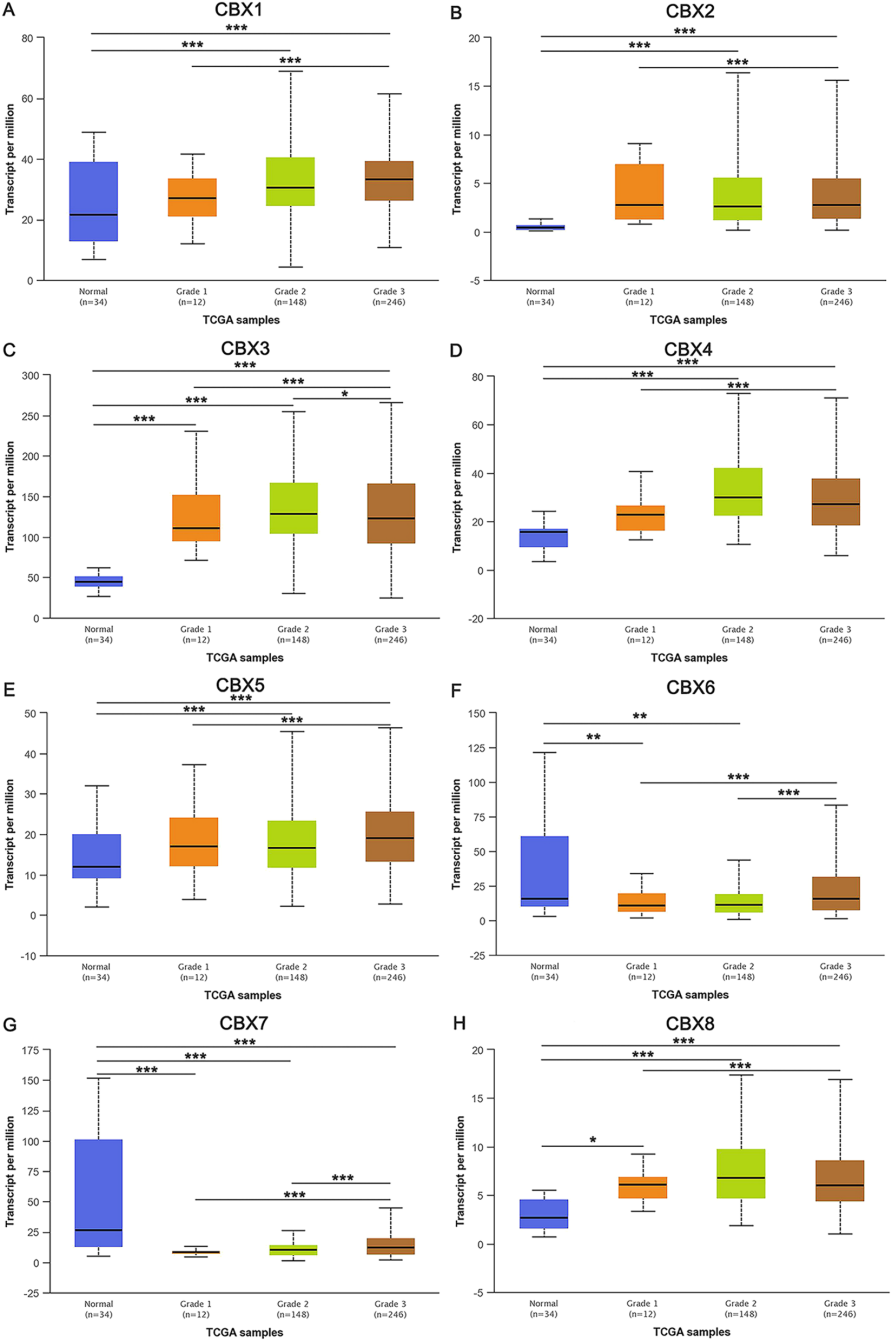
Association of the mRNA expression levels of CBXs with tumor grades in GC patients. The mRNA expression levels of 8 CBXs were significantly correlated with tumor grades, and as tumor grade increased, the mRNA expression levels of CBXs increased. The mRNA expression levels of CBX1/2/5/6/7 were the highest in grade 3 tumors (**A**,**B**,**E**–**G**). However, the mRNA expression levels of CBX3/4/8 were the highest in grade 2 tumors (**C**,**D**,**H**). *P ˂ 0.05, **P ˂ 0.01, ***P ˂ 0.001.

Associations between the mRNA expression levels of CBXs with cancer stages and grades were analyzed with the UALCAN database. As shown in Fig. 2, the results showed that when compared with normal tissues, the mRNA expression level of CBX7 was downregulated in tumor tissues with different stage, whereas CBX2/3/4/8 was upregulated. As shown in Fig. 3, the mRNA expression levels of CBX3/8 in tumor tissues with different grade were higher than that in normal tissues. The mRNA expression level of CBX7 in cancer tissues was lower than that in normal tissues. However, a consistent significant association of CBXs with tumor stage or grade could not be detected.

### Prognostic value of the mRNA expression levels of CBXs in GC patients

To explore the prognostic value of the mRNA expression levels of CBXs in GC patients, the clinical characteristics (Supplementary Table 1) and mRNA expression levels of CBXs in 335 GC patients were downloaded from the TCGA database and used to assess the prognostic value of the mRNA expression levels of CBXs for OS in GC patients by multivariate Cox survival regression analysis. The 1-year, 3-year and 5-year survival rates of GC patients were 76.5%, 46.8% and 35.3%, respectively. The results of the regression analysis showed that high mRNA expression levels of CBX3 (HR 0.613, 95% CI 0.424–0.887, *P* = 0.010) and CBX8 (HR 0.652, 95% CI 0.457–0.929, *P* = 0.018) were related to prolonged OS in GC patients, and the mRNA expression levels of CBX1 (HR 0.981, 95% CI 0.684–1.406, *P* = 0.916), CBX2 (HR 0.872, 95% CI 0.609–1.251, *P* = 0.458), CBX4 (HR 1.012, 95% CI 0.709–1.444, *P* = 0.949), CBX5 (HR 1.279, 95% CI 0.891–1.836, *P* = 0.182), CBX6 (HR 1.158, 95% CI 0.795–1.686, *P* = 0.445), and CBX7 (HR 1.170, 95% CI 0.816–1.676, *P* = 0.393) were not associated with the OS of GC patients after adjusting for age, gender, race, pharmaceutical therapy, radiation therapy, grade, stage, topography, lymph node status, and metastasis (Supplementary Tables 2–9). These results showed that the transcriptional expression levels of CBX3/8 were independent prognostic factors for OS in GC patients. Then, GSE84437 from the Gene Expression Omnibus (GEO) database was used to verify the correlation between CBX3/8 mRNA expression levels and the prognosis of GC patients. The results showed that high mRNA expression levels of CBX3 (HR 0.722, 95% CI 0.547–0.954, *P* = 0.022) and CBX8 (HR 0.688, 95% CI 0.522–0.908, *P* = 0.008) were associated with prolonged OS in GC patients (Supplementary Table 10) after adjusting for age, gender, T and N status.

### Genetic mutations in CBXs and their associations with OS and DFS

**Figure 4:**
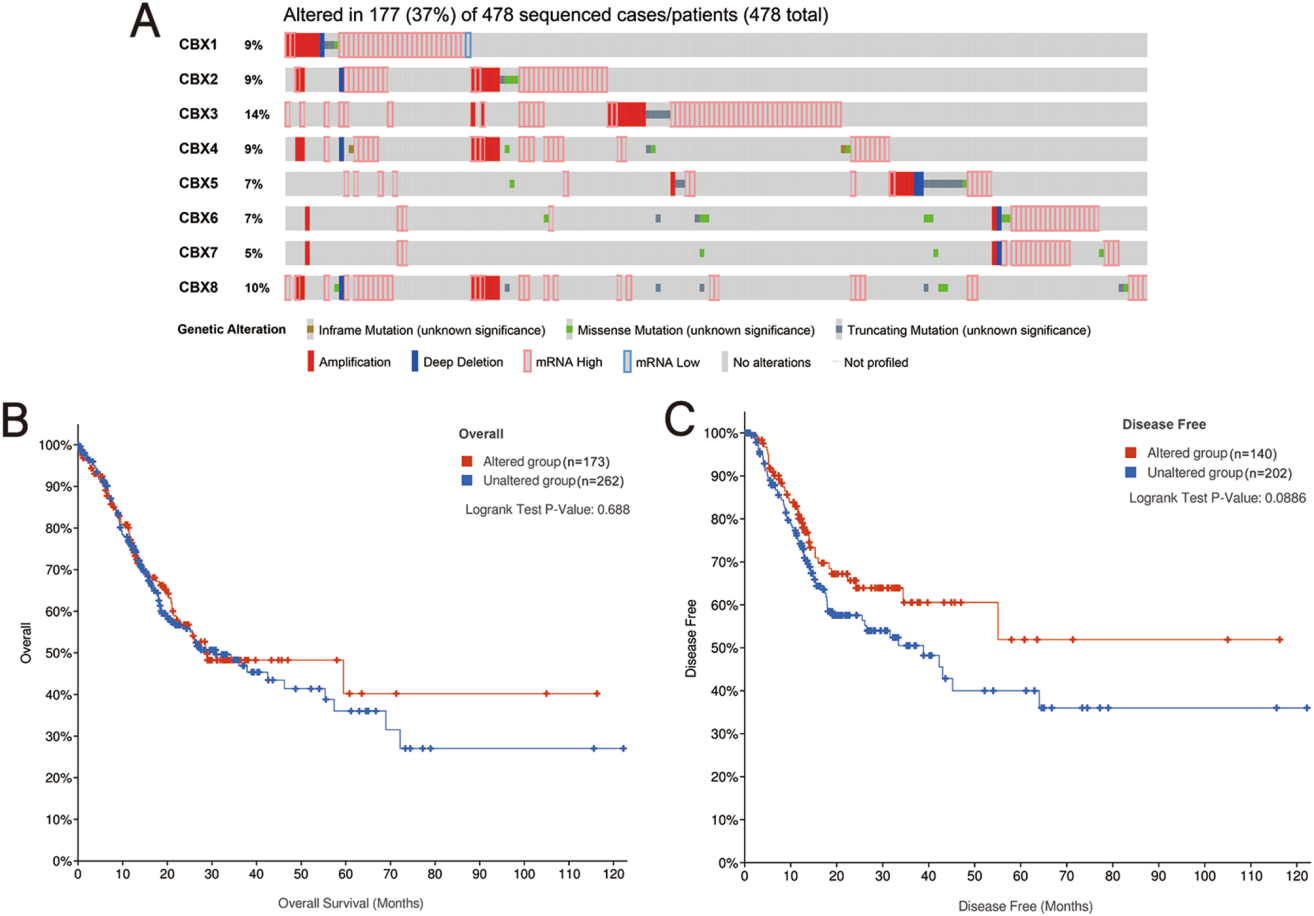
Genetic mutations in CBXs and their association with OS and DFS in GC patients (cBioPortal). The mutation rate of CBXs in GC patients was 37%. CBX3 and CBX8 were the two genes with the most genetic alterations, with mutation rates of 14% and 10%, respectively (**A**). Genetic mutations in CBXs were not associated with OS (**B**) or DFS (**C**) in GC patients. No variables were adjusted.

Mutations in CBXs genes in GC patients were analyzed with the cBioPortal online tool and the results showed that among the 478 GC patients with sequencing data, 177 had genetic alterations, with a mutation rate of 37% (Fig. 4A). CBX3 and CBX8 were the two genes with the most genetic alterations, with mutation rates 14% and 10%, respectively. The results from the Kaplan–Meier plotter and log-rank tests in the cBioPortal online tool showed that genetic alterations in CBXs (including all genetic variants of CBXs) had no effect on either OS or DFS in GC patients (OS: *P* = 0.688, Fig. 4B; DFS: *P* = 0.0886, Fig. 4C). Then, the present study explored whether a single genetic alteration in CBXs was associated with prognosis in GC patients. The results showed that none of the genetic alterations in CBX1/2/3/4/5/6/7/8 had any effects on OS (all *P* > 0.05) or DFS (all *P* > 0.05).

## Discussion

It has been reported that epigenetic regulation is involved in the development and progression of GC^34^. CBX family proteins are important components of epigenetic regulation complexes^9^. Increasing evidence has shown that CBX proteins play key roles in tumorigenesis by blocking differentiation and promoting the self-renewal of tumor stem cells^9^. Currently, among the CBX family members, CBX3 and CBX7 have been reported to be related to GC^35,36^. The roles of most CBX family members in the progression of gastric carcinoma are still unclear. In this study, we conducted a systematic and comprehensive analysis of all the CBX family members using bioinformatics methods, aiming to explore the prognostic significance of CBXs in GC.

CBX2 is a crucial component of the chromatin-regulated PRC1 complex^37^. Clermont et al. found that CBX2 was upregulated in metastatic castration-resistant prostate cancer (CRPC), and CBX2 depletion abrogated cell viability and induced caspase 3-mediated apoptosis in metastatic PCa cell lines^38^. The present study demonstrated that CBX2 production was also upregulated in gastric cancer tissues compared with normal tissues.

One study showed that the expression level of CBX3 in patients with GC and atrophic gastritis (AG) was higher than that in patients with normal gastric mucosa. As reported, CBX3 promotes colon cancer growth by directly regulating the cyclin-dependent kinase inhibitor, p21^Waf1/Cip1^ (CDKN1A)^23^. The overexpression of CBX3 is related to the progression of GC^35^. The present study showed that CBX3 expression was significantly upregulated in GC tissues compared with normal tissues, and CBX3 overexpression was correlated with prolonged OS in GC patients. This finding may be because GC patients with a high mRNA expression level of CBX3 are more sensitive to clinical treatment than those with a low mRNA expression level of CBX3. For example, Lin et al. found that overexpressed CBX3 contributes to the sensitivity of GC patients to chemotherapy. A survival advantage for the high CBX3 group compared with the low CBX3 group was observed in those who received chemotherapy^39^. The present study also showed that in GC patients receiving pharmaceutical therapy (HR 0.369, 95% CI 0.144–0.941, *P* = 0.037) and radiation therapy (HR 0.530, 95% CI 0.297–0.945, *P* = 0.031), the prognosis of the high CBX3 group was better than that of the low CBX3 group.

CBX4 plays a dual role, promoting and inhibiting carcinogenesis. Overexpressed CBX4 can evoke oncogenic activities through the Notch 1 signaling pathway in BC^40^. However, CBX4 can recruit histone deacetylase 3 (HDAC3), enable HDAC3 to bind to the Runx2 promoter, block the expression of Runx2, and inhibit the metastasis of CRC^41^. The mRNA expression level of CBX4 in liver cancer tissues was higher than that in normal tissues^42^. Luo et al. reported that the CBX4 rs77447679 polymorphism was positively associated with GC, and individuals with the CC genotype had a low risk of developing GC. The present study indicated overexpressed CBX4 in GC patients.

Overexpression of CBX5 has been observed in many cancers, such as pulmonary carcinoma, BC and PCa^13^. Claerhout et al. revealed that the mRNA expression level of CBX5 was elevated in GC tissues^43^. Since CBX5 was found to regulate the stem-like properties and aggressiveness of lung tumor stem-like cells, it might be capable of predicting the prognosis of pulmonary carcinoma^44^. Guo et al. also found that CBX5 could promote in vivo GC cell proliferation, migration, and invasion^45^. The present study showed that CBX5 mRNA expression was higher in GC tissues than in normal tissues.

CBX7 expression is the most important characteristic of CBXs in cancer-related research. Many studies found that CBX7 expression is decreased in many cancer tissues. For example, CBX7 is downregulated in pancreatic cancer and negatively regulates PTEN/Akt signal transduction during the development of pancreatic cancer^46^. In ovarian cancer, CBX7 inhibits tumor growth and metastasis by binding to E-box to inhibit the function of TWIST1^47^. Kim et al. reported that CBX7 inhibited the Wnt/β-catenin pathway by increasing the expression of the Wnt antagonist DKK-1, thereby inhibiting the occurrence of BC^48^. In addition, CBX7 expression was significantly lower in gastric, colorectal and hepatocellular carcinoma than in normal tissues^49^. Bilgic et al. observed lower CBX7 expression in the mucosa of patients with AG and GC compared with controls^35^. Ma et al. also found that the expression of CBX7 in GC tissues was significantly lower than that in normal tissues^50^. Results from both the Oncomine and UALCAN databases showed that the mRNA expression level of CBX7 was lower in GC tissues than in normal tissues. However, Kaplan–Meier plotter showed that high CBX7 mRNA expression was associated with poor OS. One explanation for this finding is that although CBX7 mRNA expression is low in cancer tissues, CBX7 protein expression is significantly elevated in tumor tissues^51^.

CBX8 might serve as an oncogene and regulate the miR-365-3p-EGR1-AKT/β-Catenin pathway. Ecotopic CBX8 production in tissues is beneficial to tumor cell growth^29^. However, high CBX8 expression is related to a low rate of tumor metastasis and a favorable prognosis in CRC patients, and the downregulated CBX8 expression inhibits CRC proliferation. Therefore, CBX8 has contradictory effects on CRC progression^52^. Ghalandary et al. found no difference in the mean CBX8 expression level between GC and adjacent normal tissues^53^. The present study also found no significant difference in CBX8 mRNA levels between GC and normal tissues, and high CBX8 expression was significantly related to improved OS in GC patients.

The increase of CBX2/3/4/5 and a decrease of CBX7 in comparison to adjacent tissues in GC may be caused by the potential differences in cellular content in tumor vs. adjacent tissues. One research on re-clustering analysis of tumor, normal and metaplastic epithelial cells from the scRNA-seq dataset revealed three subclasses^54^. The first subclass consisted of normal gastric epithelial cells—over 80% were derived from normal gastric samples. Normal epithelial cells were detected in all samples regardless of their origin from tumor, normal or metaplastic tissue. The second subclass consisted of tumor-specific epithelial cells. Approximately 98% of these cells originated from tumor samples. The third subclass involved epithelial cells derived from GC as well as normal tissue^54^. This indicated the extent of inter-tumor heterogeneity among all of the GCs, meaning that each individual tumor had distinct transcriptional properties.

This study showed that CBX3/8 were independent prognostic factors in GC patients from the TCGA database, and their significance was verified in the GSE84437 online database. This study has some limitations. First, all the analyzed data were retrieved from online databases, and further studies with larger sample sizes are necessary to validate the findings and explore the clinical application of CBXs. Second, because of the heterogeneity of the database, the CBX protein-encoded genes were not consistently evaluated. For example, in the Oncomine database, CBX5/6/7/8 were not associated with GC, which may be due to the small number of GC patients. Therefore, the results of this study need to be confirmed in a large cohort study. Finally, the present study failed to explore the potential mechanisms of CBXs in GC development. Future experimental studies are required to investigate the mechanisms involving CBXs and GC.

In conclusion, multivariate analysis suggested that high mRNA levels of CBX3/8 were independent prognostic factors for improved OS in GC patients. These results indicate that CBX3/8 could be prognostic biomarkers for the survival of GC patients.

## Supplementary Information

Supplementary Tables.
