# Supplementary material for: Evaluation of the prognostic value of CBXs in gastric cancer patients
Source: Sci Rep. 2021 Jun 11;11:12375. doi: 10.1038/s41598-021-91649-7 (PMC8196000; doi:10.1038/s41598-021-91649-7)
Supplement: Supplementary file 1 — Supplementary Tables. [file 41598_2021_91649_MOESM1_ESM.pdf]

Title page

Prognostic value of CBX3/8 in gastric cancer patients

Mengya He[1], Limin Yue[1*], Haiyan Wang[1], Feiyan Yu[1], Mingyang Yu[1], Peng Ni[1], Ke Zhang[1], Shuaiyin Chen[1], Guangcai Duan[1*], Rongguang Zhang[2,3*]

[1]Department of Epidemiology, College of Public Health, Zhengzhou University

[2]Department of Epidemiology, College of Public Health, Hainan Medical University

[3]Department of Experimentation Center, College of Public Health, Zhengzhou University

Mengya He and Limin Yue are the co-first authors; Limin Yue, Guangcai Duan and Rongguang Zhang are the co-corresponding authors.

***Corresponding Author:**

Limin Yue. Email: ylm1412217418@163.com, Department of Epidemiology, College of Public Health, Zhengzhou University, No.100 Kexue Avenue, Zhengzhou 450001, China. ORCID: 0000-0001-7035-1007.

Guangcai Duan. Email: gcduan@zzu.edu.cn, Department of Epidemiology, College of Public Health, Zhengzhou University, No.100 Kexue Avenue, Zhengzhou 450001, China. ORCID: 0000-0003-1353-9833.

Rongguang Zhang. Email: zrg@zzu.edu.cn, Department of Epidemiology, College of Public Health, Hainan Medical University, No.3 Xueyuan Road, Longhua District, Haikou 570216, China; Experimentation Center, College of Public Health, Zhengzhou University, No.100 Kexue Avenue, Zhengzhou 450001, China. ORCID: 0000-0001-9961-2434.

Supplementary Table 1. Basic characteristics of 335 GC patients (from TCGA survival data).

Variables	N (%)
Gender	
Male	209 (62.4%)
Female	126 (37.6%)
Age	
35-54	55 (16.4%)
55-74	206 (61.5%)
≥ 75	74 (22.1%)
Race	
White	214 (63.9%)
Asian	65 (19.4%)
Black or African American	10 (3.0%)
Not reported	46 (13.7%)
Pharmaceutical therapy	
No	154 (46.0%)
Yes	148 (44.2%)
Not reported	33 (9.9%)
Radiation therapy	
No	246 (73.4%)
Yes	57 (17.0%)
Not reported	32 (9.6%)
Grade	
G1	10 (2.7%)
G2	132 (36.0%)
G3	216 (58.9%)
GX	9 (2.5%)
Stage	
I-II	158 (43.1%)
III-IV	186 (50.7%)
unknown	23 (6.3%)
Topography	
T1-2	88 (26.3%)
T3-4	239 (71.3%)
TX	8 (2.4%)
Lymph Node	
N0	98 (29.3%)
N1-3	221 (66.0%)
NX	16 (4.8%)
Metastasis	
M0	299 (89.3%)
M1	23 (6.9%)
MX	13 (3.9%)

OS: overall survival, GC: gastric cancer.

Supplementary Table 2. Multivariate analysis of OS in 335 GC patients (from TCGA survival data).

Variables	Multivariate analysis		
	Hazard ratio	95% CI	*P* value
Gender			
Male	Ref		
Female	0.819	0.562-1.193	0.298
Age			
35-54	Ref		
55-74	1.438	0.831-2.487	0.194
≥ 75	1.933	1.025-3.642	0.042
Race			
White	Ref		
Asian	0.812	0.466-1.416	0.464
Black or African American	1.373	0.605-3.112	0.448
Pharmaceutical therapy			
No	Ref		
Yes	0.826	0.534-1.279	0.392
Radiation therapy			
No	Ref		
Yes	0.575	0.323-1.023	0.060
Grade			
G1	Ref		
G2	1.209	0.386-3.788	0.744
G3	1.065	0.347-3.272	0.912
Stage			
I-II	Ref		
III-IV	1.341	0.780-2.307	0.288
Topography			
T1-2	Ref		
T3-4	1.406	0.828-2.387	0.208
Lymph Node			
N0	Ref		
N1-3	1.599	0.881-2.901	0.122
Metastasis			
M0	Ref		
M1	1.617	0.925-2.826	0.091
CBX1			
Low	Ref		
High	0.981	0.684-1.406	0.916▲

OS: overall survival, GC: gastric cancer. ▲Adjusting for age, gender, race, pharmaceutical therapy, radiation therapy, grade, stage, topography, lymph node, and metastasis.

Supplementary Table 3. Multivariate analysis of OS in 335 GC patients (from TCGA survival data).

Variables	Multivariate analysis		
	Hazard ratio	95% CI	*P* value
Gender			
Male	Ref		
Female	0.818	0.561-1.192	0.295
Age			
35-54	Ref		
55-74	1.470	0.847-2.551	0.171
≥ 75	1.986	1.048-3.764	0.035
Race			
White	Ref		
Asian	0.821	0.471-1.432	0.488
Black or African American	1.376	0.608-3.114	0.444
Pharmaceutical therapy			
No	Ref		
Yes	0.826	0.534-1.279	0.392
Radiation therapy			
No	Ref		
Yes	0.836	0.539-1.297	0.423
Grade			
G1	Ref		
G2	1.241	0.393-3.920	0.713
G3	1.093	0.353-3.379	0.878
Stage			
I-II	Ref		
III-IV	1.329	0.774-2.280	0.302
Topography			
T1-2	Ref		
T3-4	1.450	0.854-2.463	0.169
Lymph Node			
N0	Ref		
N1-3	1.584	0.874-2.870	0.130
Metastasis			
M0	Ref		
M1	1.673	0.951-2.942	0.074
CBX2			
Low	Ref		
High	0.872	0.609-1.251	0.458▲

OS: overall survival, GC: gastric cancer. ▲Adjusting for age, gender, race, pharmaceutical therapy, radiation therapy, grade, stage, topography, lymph node, and metastasis.

Supplementary Table 4. Multivariate analysis of OS in 335 GC patients (from TCGA survival data).

Variables	Multivariate analysis		
	Hazard ratio	95% CI	*P* value
Gender			
Male	Ref		
Female	0.823	0.564-1.203	0.315
Age			
35-54	Ref		
55-74	1.576	0.908-2.735	0.106
≥ 75	2.063	1.097-3.880	0.025
Race			
White	Ref		
Asian	0.896	0.515-1.559	0.697
Black or African American	1.521	0.674-3.430	0.312
Pharmaceutical therapy			
No	Ref		
Yes	0.679	0.474-0.972	0.034
Radiation therapy			
No	Ref		
Yes	0.530	0.297-0.945	0.031
Grade			
G1	Ref		
G2	1.190	0.375-3.770	0.768
G3	1.055	0.338-3.296	0.926
Stage			
I-II	Ref		
III-IV	1.295	0.752-2.228	0.351
Topography			
T1-2	Ref		
T3-4	1.588	0.936-2.693	0.086
Lymph Node			
N0	Ref		
N1-3	1.552	0.854-2.820	0.150
Metastasis			
M0	Ref		
M1	1.582	0.906-2.762	0.107
CBX3			
Low	Ref		
High	0.613	0.424-0.887	0.010▲

OS: overall survival, GC: gastric cancer. ▲Adjusting for age, gender, race, pharmaceutical therapy, radiation therapy, grade, stage, topography, lymph node, and metastasis.

Supplementary Table 5. Multivariate analysis of OS in 335 GC patients (from TCGA survival data).

Variables	Multivariate analysis		
	Hazard ratio	95% CI	*P* value
Gender			
Male	Ref		
Female	0.819	0.562-1.193	0.298
Age			
35-54	Ref		
55-74	1.435	0.827-2.491	0.199
≥ 75	1.930	1.023-3.640	0.042
Race			
White	Ref		
Asian	0.810	0.466-1.409	0.456
Black or African American	1.370	0.605-3.103	0.451
Pharmaceutical therapy			
No	Ref		
Yes	0.826	0.534-1.279	0.392
Radiation therapy			
No	Ref		
Yes	0.577	0.324-1.026	0.061
Grade			
G1	Ref		
G2	1.204	0.385-3.764	0.750
G3	1.064	0.347-3.268	0.913
Stage			
I-II	Ref		
III-IV	1.339	0.779-2.299	0.291
Topography			
T1-2	Ref		
T3-4	1.411	0.836-2.383	0.198
Lymph Node			
N0	Ref		
N1-3	1.601	0.882-2.904	0.121
Metastasis			
M0	Ref		
M1	1.617	0.924-2.828	0.092
CBX4			
Low	Ref		
High	1.012	0.709-1.444	0.949▲

OS: overall survival, GC: gastric cancer. ▲Adjusting for age, gender, race, pharmaceutical therapy, radiation therapy, grade, stage, topography, lymph node, and metastasis.

Supplementary Table 6. Multivariate analysis of OS in 335 GC patients (from TCGA survival data).

Variables	Multivariate analysis		
	Hazard ratio	95% CI	*P* value
Gender			
Male	Ref		
Female	0.804	0.551-1.171	0.255
Age			
35-54	Ref		
55-74	1.395	0.805-2.416	0.235
≥ 75	1.884	0.999-3.552	0.050
Race			
White	Ref		
Asian	0.826	0.474-1.438	0.499
Black or African American	1.251	0.547-2.862	0.595
Pharmaceutical therapy			
No	Ref		
Yes	0.799	0.513-1.243	0.319
Radiation therapy			
No	Ref		
Yes	0.586	0.329-1.047	0.071
Grade			
G1	Ref		
G2	1.240	0.394-3.898	0.713
G3	1.107	0.357-3.431	0.860
Stage			
I-II	Ref		
III-IV	1.309	0.759-2.257	0.334
Topography			
T1-2	Ref		
T3-4	1.420	0.840-2.398	0.190
Lymph Node			
N0	Ref		
N1-3	1.621	0.891-2.951	0.114
Metastasis			
M0	Ref		
M1	1.588	0.908-2.778	0.105
CBX5			
Low	Ref		
High	1.279	0.891-1.836	0.182▲

OS: overall survival, GC: gastric cancer. ▲Adjusting for age, gender, race, pharmaceutical therapy, radiation therapy, grade, stage, topography, lymph node, and metastasis.

Supplementary Table 7. Multivariate analysis of OS in 335 GC patients (from TCGA survival data).

Variables	Multivariate analysis		
	Hazard ratio	95% CI	*P* value
Gender			
Male	Ref		
Female	0.821	0.563-1.196	0.304
Age			
35-54	Ref		
55-74	1.428	0.827-2.467	0.202
≥ 75	1.934	1.026-3.645	0.041
Race			
White	Ref		
Asian	0.820	0.472-1.425	0.482
Black or African American	1.358	0.599-3.078	0.464
Pharmaceutical therapy			
No	Ref		
Yes	0.834	0.539-1.291	0.416
Radiation therapy			
No	Ref		
Yes	0.589	0.331-1.049	0.072
Grade			
G1	Ref		
G2	1.159	0.370-3.633	0.800
G3	1.024	0.332-3.157	0.967
Stage			
I-II	Ref		
III-IV	1.361	0.793-2.335	0.263
Topography			
T1-2	Ref		
T3-4	1.412	0.837-2.383	0.196
Lymph Node			
N0	Ref		
N1-3	1.566	0.862-2.845	0.141
Metastasis			
M0	Ref		
M1	1.592	0.911-2.782	0.103
CBX6			
Low	Ref		
High	1.158	0.795-1.686	0.445▲

OS: overall survival, GC: gastric cancer. ▲Adjusting for age, gender, race, pharmaceutical therapy, radiation therapy, grade, stage, topography, lymph node, and metastasis.

Supplementary Table 8. Multivariate analysis of OS in 335 GC patients (from TCGA survival data).

Variables	Multivariate analysis		
	Hazard ratio	95% CI	*P* value
Gender			
Male	Ref		
Female	0.826	0.567-1.205	0.321
Age			
35-54	Ref		
55-74	1.441	0.835-2.489	0.190
≥ 75	1.927	1.024-3.627	0.042
Race			
White	Ref		
Asian	0.832	0.477-1.451	0.518
Black or African American	1.347	0.594-3.054	0.476
Pharmaceutical therapy			
No	Ref		
Yes	0.829	0.536-1.283	0.401
Radiation therapy			
No	Ref		
Yes	0.567	0.319-1.009	0.054
Grade			
G1	Ref		
G2	1.132	0.360-3.560	0.832
G3	1.012	0.328-3.120	0.983
Stage			
I-II	Ref		
III-IV	1.351	0.788-2.317	0.274
Topography			
T1-2	Ref		
T3-4	1.436	0.848-2.433	0.178
Lymph Node			
N0	Ref		
N1-3	1.595	0.879-2.895	0.124
Metastasis			
M0	Ref		
M1	1.649	0.944-2.880	0.079
CBX7			
Low	Ref		
High	1.170	0.816-1.676	0.393▲

OS: overall survival, GC: gastric cancer. ▲Adjusting for age, gender, race, pharmaceutical therapy, radiation therapy, grade, stage, topography, lymph node, and metastasis.

Supplementary Table 9. Multivariate analysis of OS in 335 GC patients (from TCGA survival data).

Variables	Multivariate analysis		
	Hazard ratio	95% CI	*P* value
Gender			
Male	Ref		
Female	0.826	0.567-1.205	0.321
Age			
35-54	Ref		
55-74	1.441	0.835-2.489	0.190
≥ 75	1.927	1.024-3.627	0.042
Race			
White	Ref		
Asian	0.832	0.477-1.451	0.518
Black or African American	1.347	0.594-3.054	0.476
Pharmaceutical therapy			
No	Ref		
Yes	0.829	0.536-1.283	0.401
Radiation therapy			
No	Ref		
Yes	0.567	0.319-1.009	0.054
Grade			
G1	Ref		
G2	1.132	0.360-3.560	0.832
G3	1.012	0.328-3.120	0.983
Stage			
I-II	Ref		
III-IV	1.351	0.788-2.317	0.274
Topography			
T1-2	Ref		
T3-4	1.436	0.848-2.433	0.178
Lymph Node			
N0	Ref		
N1-3	1.595	0.879-2.895	0.124
Metastasis			
M0	Ref		
M1	1.649	0.944-2.880	0.079
CBX8			
Low	Ref		
High	0.652	0.457-0.929	0.018▲

OS: overall survival, GC: gastric cancer. ▲Adjusting for age, gender, race, pharmaceutical therapy, radiation therapy, grade, stage, topography, lymph node, and metastasis.

Supplementary Table 10. Association of CBX3/8 with OS in 433 GC patients (from GEO database GSE84437).

Variables	Multivariate analysis		
	Hazard ratio	95% CI	*P* value
CBX3			
Low	Ref		
High	0.722	0.547-0.954	0.022▼
CBX8			
Low	Ref		
High	0.688	0.522-0.908	0.008▼

OS: overall survival, GC: gastric cancer. ▼Adjusting for age, gender, T and N status.
